# Dietary tryptophan restriction selectively suppresses lipogenesis, lowers cholesterol, and reduces adiposity without altering food consumption

**DOI:** 10.21203/rs.3.rs-10855823/v1

**Published:** 2026-10-12

**Authors:** Maralice Conacci-Sorrell, pedro Augusto silva Nogueira, Lizbeth Perez-Castro, Niranjan Venkateswarran, Emily Parks, Kathleen Mannino, Yi-Heng Hao, Igor dos Santos, Ruth Gordillo, Jessica Kilgore, Isabella DePasquale, Joyane Eriom, Lei Dong, Lin Xu, Andrew Lemoff, Ralph DeBerardinis, Noelle Williams, Philipp Scherer

## Abstract

We interrogated the consequences of removing individually each of the nine essential amino acids using defined diets in mice. Among the essential amino acids, only tryptophan (Trp) depletion induced rapid and selective fat loss with minimal effects on lean body mass while retaining normal feeding behavior. We named this intervention “Trypt-out.” Trypt-out prevented and reversed obesity, hepatic steatosis, and glucose intolerance in both leptin-deficient and diet-induced obesity. Trypt-out also remained highly effective even with obesogenic high-fat and high-sucrose diets. Mechanistically, short-term Trp depletion did not impair global protein synthesis but selectively resulted in profound inhibition of lipogenic and cholesterol biosynthetic enzymes. This resulted in reduction in cholesterol that exceeded the effects of statin therapy. Trp as a master regulator of systemic lipid homeostasis and establish Trypt-out as a well-tolerated, rapid- acting dietary strategy to selectively eliminate adiposity, normalize metabolic dysfunction, and lower cholesterol without reducing food consumption or lean mass.

